# SpO_2_/FiO_2_ ratio as a better metric for assessment of RBC transfusion effectiveness in non-traumatic critically ill patients with physiologic derangements

**DOI:** 10.1371/journal.pone.0327537

**Published:** 2025-07-03

**Authors:** Tilendra Choudhary, Geoffrey Smith, John D. Roback, Ravi M. Patel, Cassandra D. Josephson, Rishikesan Kamaleswaran

**Affiliations:** 1 Department of Surgery, Duke University School of Medicine, Durham, North Carolina, United States of America; 2 Department of Anesthesiology, Duke University School of Medicine, Durham, North Carolina, United States of America,; 3 Department of Pathology and Laboratory Medicine, Emory University School of Medicine, Atlanta, Georgia, United States of America; 4 Department of Pediatrics, Emory University School of Medicine, Atlanta, Georgia, United States of America,; 5 Department of Oncology, The Johns Hopkins University School of Medicine, Baltimore, Maryland, United States of America; 6 Cancer and Blood Disorders Institute, Johns Hopkins All Children’s Hospital, St. Petersburg, Florida, United States of America; Pescara General Hospital, ITALY

## Abstract

Identifying critically ill patients who are likely to improve their respiratory physiology following RBC transfusion is dynamic and difficult. Current decision tools are over-reliant on hemoglobin transfusion thresholds, without considering respiratory measures that may reflect physiologic effects of anemia and functional responses to RBC transfusion. Further, routine clinical measures to determine transfusion efficacy beyond hemoglobin increment are lacking to identify patients as responders or non-responders. We present a two-center retrospective cohort study aiming to determine a potential biomarker to assess the physiologic response of RBC transfusion for non-traumatic ICU patients. The study was performed with 13,274 eligible patients at the first center. Another 3,757 from the second center were used as a validation population. We introduced a comparative analysis of two respiratory measures, SpO_2_ and SpO_2_/FiO_2_ (SF) ratio, in addition to hemoglobin, to assess individual patient responses to RBC transfusion. A statistical study was performed to compare these markers before and after the transfusion interval. Based on quantitative statistical analyses, we found SF ratio to be a more effective biomarker than hemoglobin alone for revealing RBC transfusion efficacy. There existed an inverse correlation between pre-transfusion SF and transfusion efficacy. The results were consistent across both centers, revealing generalizability. With the SF data from both the centers, we also developed a random forest-based regression model that significantly evaluated the level of transfusion effectiveness (*p* < 0.001).

## Introduction

Critical ill patients in intensive care units (ICU) often develop anemia and coagulopathy that are associated with adverse outcomes, such as high risk of life-threatening scenarios, thrombosis, and coronary artery diseases. To treat the anemia and coagulopathy following surgery, trauma or medical conditions, patients have been transfused with red blood cells (RBCs) for decades [1,2]. Annually about 85 million RBC units are used for transfusion worldwide, and approximately 15 million are transfused every year in the United States only [3]. A major physical damage, injury or massive bleeding due to an accident or hemorrhage is typically referred as *Trauma*. Massive blood transfusions (MTs) are sometimes essential for traumatic patients. In contrast, reasons for non-traumatic blood transfusions include healthy blood cell deficiency (e.g., anemia, thrombocytopenia, leukopenia), coagulopathy, and other health disorders (e.g., hemophilia, kidney or liver disease, severe infection, and sickle cell disease). An assessment of transfusion response and transfusion necessity in non-traumatic ICU patients is more difficult than in traumatic patients. The hallmark trigger for initiating transfusions has been the criterion of hemoglobin (Hb) ≤7 g/dL [1,2,4]. However, a significant percentage of transfusions performed are often identified as inappropriate [5,6], thus contributing to adverse events such as acute immune hemolytic reactions, bloodborne infections, transfusion associated circulatory overload (TACO) and transfusion-related acute lung injury (TRALI) [7–9]. There has been much interest in the discovery of alternative biomarkers that may indicate a quantitative utility of RBC transfusions. The goal of our current study was to identify generalized potential markers to evaluate RBC transfusion efficacy especially in critical illnesses in support of hemoglobin-based thresholding approach. SpO_2_ represents the saturation of oxygen in the patient’s blood expressed as a percentage, whereas FiO_2_ indicates fractional inspiration of oxygen, the amount of oxygen that is inhaled. One potential non-invasive derived measure is the SpO_2_/FiO_2_ (SF) ratio, which incorporates blood oxygen content along with oxygen concentration in gas exchange. This equation couples the amount of oxygen mixed with the air the patient is inhaling (FiO_2_) with the degree of hypoxemia (SpO_2_) the patient is experiencing. FiO_2_ is a measure of inhaled atmospheric/supplemental oxygen content and can be titrated according to the SpO_2_ measure. When oxygen supply and its consumption are incongruent, cells are damaged and subsequently die [10]. For instance, in chronic obstructive pulmonary disease (COPD), supplemental oxygen is recommended to start when the SpO_2_ falls below 88% [10]. Clinically, SF < 144 is marked severe, 144–235 moderate, 235–315 mild, and >315 normal [11]. Based on the potential for the SF ratio to provide a functional measure of oxygen requirements and delivery, the SF was hypothesized to be a more accurate biomarker for RBC transfusion response. This study endeavored to investigate the effect of RBC transfusion on SF ratio in a cohort of critically ill patients to determine the physiologic response to RBC transfusion. The study may help in devising decision-making tools, data benchmarking, and future research to optimize treatment strategies for RBC transfusion of ICU patients.

## Materials and methods

This study was reviewed and approved by the Emory University IRB (No. STUDY00000302) as human subjects, and waiver of informed consent was obtained. All procedures were followed in accordance with the ethical standards of the responsible committee on human experimentation (institutional/regional) and with the Helsinki Declaration of 1975. Continuous physiological data were archived using the BedMaster (Excel Medical, Jupiter, FL) software. SpO_2_ was collected at 1-hour sampling interval and used to derive the SF ratio continuously from admission through to discharge for patients. Hemoglobin and FiO_2_ values were derived from the electronic medical record for enrolled patients. The retrospective data was lastly accessed on Mar. 30^th^, 2024, for this research. The authors had also access to information that could identify individual participants during or after data collection.

In this article, we present a multi-center retrospective cohort study conducted at two high volume academic hospitals located in the southeastern U.S.A. Adult non-traumatic ICU patients (≥18 years) admitted to either of these two metropolitan hospitals, Emory University Hospital and Grady Memorial Hospital (Atlanta, GA) anytime in 2016–2020, who received RBC transfusion, were eligible for enrollment in the study. We adopted a multi-center derivation and validation study design by first establishing relationship of SF and transfusion response using data from Emory University Hospital, and then validating this relationship against data collected from Grady Memorial Hospital. Patients were excluded for the following reasons: a) if they had trauma and high bleeding injury who received massive transfusions, b) if they did not have continuous monitoring data archived during encounter, c) were discharged or died after ICU admission within 24 hours, due to limited duration of monitoring data available; and, d) who did not have continuous physiological data up to 24 hours before and after the transfusion. We also excluded successive RBC transfusion episodes occurred within next 24 hours of an RBC transfusion event to avoid data overlapping. Inclusion of patients was not limited for any specific diagnosis.

**Fig 1:**
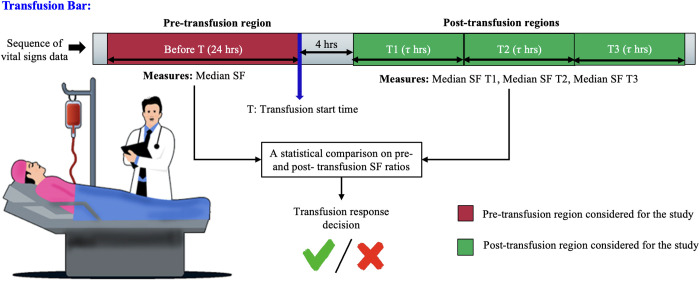
Overview of the study design. One pre-transfusion period of 24 hours and three post-transfusion periods of 9 hours each were used to analyze short-term vs. sustained physiological improvements.


We divided the timing of RBC transfusion into two windows, the period before the initiation of RBC transfusion and the period after its completion. Specifically, 24-hour data sequences were considered for the period before transfusion, and three subsequent post-RBC transfusion periods (T1, T2, T3; 9-hour duration each) were evaluated for the post-transfusion period, typically following a 4-hour period of RBC infusion. In the study, we wanted to assess early response (less than or equal to 12 hours) and delayed response (after 24 hours) of transfusion. Thus, three post-transfusion periods (T1, T2, T3) of each 9 hours interval were chosen empirically. We applied median aggregation to the data of our cohort, across the pre-transfusion window and post-transfusion time-windows. An overview of our method is shown in Fig 1. The reason for the selection of the three periods was to identify short-term vs. sustained improvements in respiratory physiology. Responses to RBC transfusions were characterized by SF differences (ΔSF) that were calculated between post- and pre-transfusion periods, considering four different ranges of baseline SF measurements (1–143, 144–235, 236–315, > 315). An increase in the SF ratio indicated improvement in respiratory physiology (either from an SpO_2_ improvement or an FiO_2_ decrease). We further employed a polynomial curve fitting scheme to evaluate the relationship between baseline SF and ΔSF, demonstrating which patient groups were more benefitted from transfusion. The following third-order polynomial function was used to model this relation in a least square sense.

$$f(x)=px^{3}+qx^{2}+rx+s$$

where p, q, r, s denote the modelling parameters. Average model coefficients with their 95% confidence intervals were evaluated after 50 randomized bootstrap sample runs for ensuring consistency in modeled curves to reduce verification bias in the study. The association between ΔSF and difference in SpO_2_ (ΔSpO_2_) was also evaluated using the Bland Altman analysis. Also, a random forest-based regression model was developed with both the hospital data for further evaluating the level of transfusion effectiveness. The SF data from both the centers were merged and sliced in for build (60%), test (20%) and validation (20%).

To evaluate the statistical significance (*p*-value) among four baseline SF groups, Chi-squared and One-way ANOVA tests were used for categorical and numeric features, respectively. Whereas Pearson correlation coefficients (r) and limit of agreements (LoA) were used to evaluate the correlation and agreement along with the significance level. Further, R-squared (R^2^), mean squared error (MSE) and mean absolute error (MAE) were used for performance assessment of the regression model.

## Results

We retrospectively evaluated a total of 82,767 patient admissions from 64,962 unique patients who were admitted to Emory ICUs from 2016–2020, of which 15,898 (~20%) received at least one RBC transfusion with no massive transfusions. Among these, 14,765 had continuous physiologic bedside monitoring data within the temporal period close to the index RBC transfusion event. After excluding patients with missing physiological data, 13,274 were selected for the analysis and a total of 18,716 transfusion episodes were noted for which the baseline SF is not missing. Our cohort consists of transfused patients across a wide range of demographic features, such as age (mean: 61.4 ± 16 years), sex (male: 48.1%), and race (Caucasian: 46.6%, African American: 45%). Similarly, from the Grady Hospital, a total of 6,375 transfusion episodes from 3,757 patient encounters were included in the study. Clinical characteristics and demographics of transfusion patients from both the centers are listed in Tables 1 and 2 for their pre-transfusion observational data.

**Table 1:** Clinical characteristics and demographic variables of the derivation dataset collected from Emory admitted RBC transfused patients based on their pre-transfusion aggregated features in four baseline SF groups – A: 1-143, B: 144-235, C: 236-315, D: > 315.

Features		Grouped by Baseline SF groups					P-value
		Whole cohort	A	B	C	D	
Transfusions, n		18716	1927	2724	3270	10795	–
Patient encounters, n		13274	1772	2306	2565	7938	–
Age in years, mean (SD)		61.4 (16.0)	62.0 (15.5)	60.7 (15.2)	61.2 (15.0)	61.5 (16.5)	0.03
Sex, n (%)	Female	9708 (51.9)	956 (49.6)	1403 (51.5)	1665 (50.9)	5684 (52.7)	0.047
	Male	9008 (48.1)	971 (50.4)	1321 (48.5)	1605 (49.1)	5111 (47.3)	
Race, n (%)	African American	8428 (45.0)	769 (39.9)	1002 (36.8)	1469 (44.9)	5188 (48.1)	<0.001
	Caucasian	8724 (46.6)	939 (48.7)	1455 (53.4)	1503 (46.0)	4827 (44.7)	
	Others	1564 (8.4)	219 (11.4)	267 (9.8)	298 (9.1)	780 (7.2)	
Ethnicity, n (%)	Hispanic	574 (3.1)	58 (3.0)	105 (3.9)	95 (2.9)	316 (2.9)	<0.001
	Non-Hispanic	16801 (89.8)	1677 (87.0)	2402 (88.2)	2882 (88.1)	9840 (91.2)	
	Others	1341 (7.2)	192 (10.0)	217 (8.0)	293 (9.0)	639 (5.9)	
Weight in kg, mean (SD)		81.8 (61.8)	82.6 (25.8)	87.9 (149.6)	87.3 (27.3)	78.5 (24.1)	<0.001
Height in cm, mean (SD)		169.1 (13.0)	169.2 (14.4)	169.4 (13.1)	169.1 (12.1)	169.1 (12.9)	0.736
Hematocrit, mean (SD)		24.5 (4.3)	27.0 (5.9)	26.2 (4.7)	24.3 (3.2)	23.7 (3.8)	<0.001
Hemoglobin, mean (SD)		7.8 (1.4)	8.7 (2.0)	8.4 (1.6)	7.8 (1.1)	7.6 (1.2)	<0.001
BUN, mean (SD)		33.7 (27.0)	32.4 (23.3)	33.0 (25.9)	35.8 (27.6)	33.4 (27.6)	<0.001
Creatinine, mean (SD)		2.2 (2.6)	1.9 (2.1)	1.9 (2.1)	2.1 (2.3)	2.3 (2.9)	<0.001
Platelets, mean (SD)		200.2 (139.8)	193.6 (132.2)	198.3 (134.3)	189.8 (132.9)	204.8 (144.2)	<0.001
Bilirubin total, mean (SD)		1.7 (4.1)	1.8 (4.6)	1.8 (4.0)	1.7 (3.8)	1.6 (4.1)	0.155
INR, mean (SD)		1.5 (0.9)	1.5 (0.9)	1.5 (0.8)	1.4 (0.8)	1.5 (0.9)	0.005
FiO_2_, mean (SD)		0.4 (0.4)	0.6 (0.3)	0.5 (0.1)	0.4 (0.0)	0.3 (0.7)	<0.001
SF ratio, mean (SD)		271.8 (99.4)	184.0 (81.6)	211.1 (54.3)	256.9 (28.2)	384.6 (75.8)	<0.001
PF ratio, mean (SD)		306.4 (143.2)	239.0 (143.7)	265.5 (127.2)	310.6 (125.1)	387.8 (137.2)	<0.001
GCS total, mean (SD)		13.5 (2.9)	12.6 (3.9)	12.4 (3.7)	12.3 (3.4)	14.2 (1.9)	<0.001
SOFA max total, mean (SD)		4.6 (3.7)	6.6 (4.7)	6.4 (4.1)	6.0 (3.6)	3.4 (2.8)	<0.001

For clinical variables, this table lists the mean and standard deviation (SD) for each baseline SF group as well as for the whole cohort. The p-value is also provided for each variable to indicate the statistical significance of the differences among the groups. For evaluating statistical significance, One-way ANOVA test was performed for continuous variables and Chi-squared test was used for categorical variables. **Abbreviations used** — n: count, mean: average, SD: standard deviation, %: percentage, PF: PaO2/FiO2 ratio, SF: SpO2/FiO2 ratio (where PaO2: partial pressure of oxygen, SpO2: peripheral oxygen saturation level, FiO2: fraction of inspired oxygen), BUN: blood urea nitrogen, INR: international normalized ratio, SOFA: sequential organ failure assessment, GCS: Glasgow coma scale. **Measurement units** — PF ratio: mmHg; SF ratio and FiO2: unitless; creatinine and bilirubin total: mg/dL; platelets: × 103/µL; hematocrit: %, hemoglobin: g/dL; BNP: pg/mL; BUN: mg/dL.

**Table 2:** Clinical characteristics and demographic variables of the validation dataset collected from Grady admitted RBC transfused patients based on their pre-transfusion aggregated features in four baseline SF groups – A: 1-143, B: 144-235, C: 236-315, D: > 315.

Features		Grouped by Baseline SF groups					P-value
		Whole cohort	A	B	C	D	
Transfusions, n		6375	689	817	2787	2082	–
Patient encounters, n		3757	622	659	1798	1554	–
Age in years, mean (SD)		53.7 (17.5)	53.8 (17.3)	55.3 (16.9)	53.5 (17.3)	53.2 (17.9)	0.037
Sex, n (%)	Female	2634 (41.3)	266 (38.6)	313 (38.3)	1136 (40.8)	919 (44.1)	0.006
	Male	3741 (58.7)	423 (61.4)	504 (61.7)	1651 (59.2)	1163 (55.9)	
Race, n (%)	African American	4751 (74.5)	497 (72.1)	533 (65.2)	2094 (75.1)	1627 (78.1)	<0.001
	Caucasian	498 (7.8)	51 (7.4)	78 (9.5)	217 (7.8)	152 (7.3)	
	Others	1126 (17.7)	141 (20.5)	206 (25.2)	476 (17.1)	303 (14.6)	
Ethnicity, n (%)	Hispanic	279 (4.4)	23 (3.3)	40 (4.9)	127 (4.6)	89 (4.3)	0.682
	Non-Hispanic	6043 (94.8)	662 (96.1)	768 (94.0)	2638 (94.7)	1975 (94.9)	
	Others	53 (0.8)	4 (0.6)	9 (1.1)	22 (0.8)	18 (0.9)	
Weight in kg, mean (SD)		81.0 (25.8)	82.9 (25.0)	84.7 (26.2)	82.1 (27.8)	77.4 (22.5)	<0.001
Height in cm, mean (SD)		171.7 (11.5)	172.0 (10.9)	172.1 (13.6)	171.8 (11.2)	171.2 (11.3)	0.133
Hematocrit, mean (SD)		24.0 (5.2)	26.3 (7.0)	25.1 (5.7)	23.5 (4.2)	23.4 (5.2)	<0.001
Hemoglobin, mean (SD)		8.0 (1.8)	8.7 (2.4)	8.5 (1.9)	7.8 (1.5)	7.8 (1.8)	<0.001
BUN, mean (SD)		31.9 (27.9)	35.8 (30.6)	34.9 (26.0)	32.8 (26.9)	28.2 (28.7)	<0.001
Creatinine, mean (SD)		2.0 (2.6)	2.3 (2.7)	2.0 (2.2)	1.9 (2.1)	2.1 (3.1)	0.009
Platelets, mean (SD)		225.0 (148.8)	213.5 (135.6)	210.7 (139.0)	222.6 (150.4)	238.1 (153.7)	<0.001
Bilirubin total, mean (SD)		1.3 (2.6)	1.7 (3.5)	1.5 (2.9)	1.2 (2.4)	1.1 (2.4)	<0.001
INR, mean (SD)		1.4 (0.8)	1.4 (0.9)	1.4 (0.9)	1.3 (0.6)	1.3 (0.9)	0.014
FiO_2_, mean (SD)		0.4 (0.2)	0.7 (0.3)	0.5 (0.1)	0.4 (0.1)	0.3 (0.1)	<0.001
SF ratio, mean (SD)		251.8 (79.9)	163.8 (75.1)	198.5 (35.7)	266.4 (56.1)	352.9 (67.4)	<0.001
PF ratio, mean (SD)		302.2 (123.8)	237.7 (123.1)	254.6 (102.1)	323.3 (112.8)	361.9 (147.4)	<0.001
GCS total, mean (SD)		11.9 (3.8)	10.5 (4.7)	9.8 (3.8)	11.0 (3.8)	14.2 (1.9)	<0.001
SOFA max total, mean (SD)		5.3 (3.9)	8.0 (4.5)	8.1 (3.9)	5.6 (3.5)	2.9 (2.5)	<0.001

For clinical variables, this table lists the mean and standard deviation (SD) for each baseline SF group as well as for the whole cohort. The p-value is also provided for each variable to indicate the statistical significance of the differences among the groups. For evaluating statistical significance, One-way ANOVA test was performed for continuous variables and Chi-squared test was used for categorical variables. **Abbreviations used —** n: count, mean: average, SD: standard deviation, %: percentage, PF: PaO_2_/FiO_2_ ratio, SF: SpO_2_/FiO_2_ ratio (where PaO_2_: partial pressure of oxygen, SpO_2_: peripheral oxygen saturation level, FiO_2_: fraction of inspired oxygen), BUN: blood urea nitrogen, INR: international normalized ratio, SOFA: sequential organ failure assessment, GCS: Glasgow coma scale. **Measurement units —** PF ratio: mmHg; SF ratio and FiO_2_: unitless; creatinine and bilirubin total: mg/dL; platelets: × 10^3^/µL; hematocrit: %, hemoglobin: g/dL; BNP: pg/mL; BUN: mg/dL.

**Fig 2:**
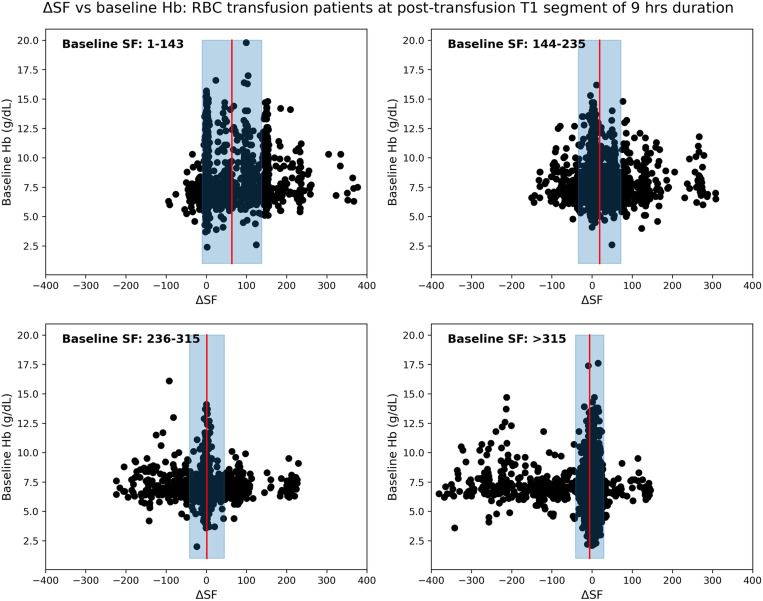
Localized study on Emory hospital patients having different ranges of baseline SF ratios. ΔSF vs baseline Hb for first post-transfusion region T1 of duration 9 hours with baseline SF range (a) 1-143, (b) 144-235, (c) 236-315, and (d) >315. Note that data-points denote the distribution of patients. Red vertical line with blueish filled region around it represents the mean ± standard deviation of the SF difference.

For the Emory data as a derivation dataset, Table 1 lists distribution of transfused patients in four groups according to their baseline SF values as A: 1−143, B: 144−235, C: 236−315, D: > 315. Initially, we attempted to establish a relation between post-transfusion SF variation and baseline Hb for four different baseline SF ranges with the derivation data. For the first post-transfusion segment T1, the relationship between ΔSF and baseline Hb is presented for four different baseline SF groups in Fig 2, representing in mean ± standard deviation of the SF difference. We found that patients with the lowest baseline SF ratio (1−143), reflecting those most critically ill, consistently demonstrated an improvement in SF ratio subsequent to RBC transfusion with a mean increase of 63.9 (95% CI 60.4–67.4) after T1, 90.3 (95% CI 86.6–94.0) after T2, 103.6 (95% CI 99.8–107.5) after T3 (Supplemental eFig 1 in S1 File). This improved effect was present across all three time periods (T1-T3), and independent of baseline Hb values (e.g., R^2^ of −0.49, −0.96 and −1.29 for T1, T2 and T3, respectively, top row of Supplemental eFig 1 in S1 File). For patients with a baseline SF > 315, reflecting the population with the least respiratory compromise, no significant improvement in SF ratio was observed following RBC transfusion, and in fact respiratory physiology worsened after transfusion (an average decrease of 5.7 (95% CI 5.0–6.4) after T1, 7.1 (95% CI 6.4–7.9) after T2, and 8.3 (95% CI 6.3–7.9) after T3), with mean differences actually reflecting worsening of respiratory physiology. Again, differences in SF ratios were not associated with pre-transfusion Hb values (e.g., R^2^ of −0.13, −0.14 and −0.15 for T1, T2 and T3, respectively, bottom row of Supplemental eFig 1 in S1 File). Patients with more moderate baseline SF ratios had more heterogeneous responses to RBC transfusions with both positive and negative SF ratios (Supplemental eFig 1 in S1 File: 2nd and 3rd rows).

**Fig 3:**
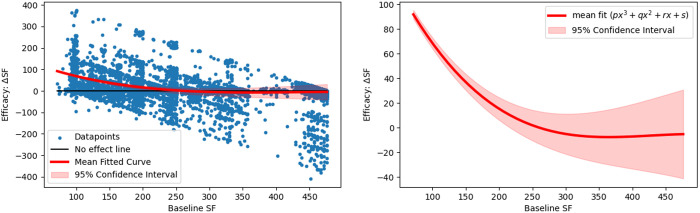
Derivation data: Relationship between baseline SF (BSF) and transfusion efficacy (ΔSF). Resultant mean curve (solid red) and its 95% confidence interval (shaded red region) modelled with a cubic-polynomial curve fitting demonstrates an inverse relationship of BSF and ΔSF. Modeling parameters of mean fitted curve are as follows: −0.000002, 0.0031, −1.28, 168.5.

**Fig 4:**
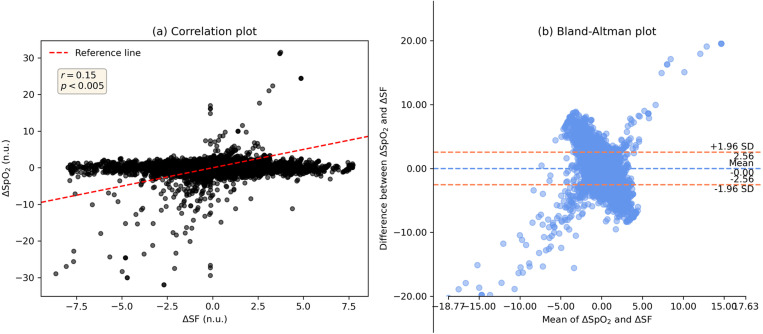
Bland Altman analysis of normalized ΔSF and ΔSpO_2_ on the derivation set. (a) correlation plot, and (b) Bland-Altman plot.

We also evaluated the relationship of baseline SF with RBC transfusion efficacy, expressed by ΔSF measures, using a polynomial curve fitting scheme. In this analysis, ΔSF is measured as difference in SF from pre-transfusion to first post-transfusion segment. The modelled resultant curve establishes an inverse relationship of baseline SF and ΔSF, demonstrating a great transfusion benefit for patients having lower baseline SFs and the effect reduces for increasing baseline SF measure. It is shown in Fig 3 with mean fitted curve and its 95% CI after a 50 randomized bootstrap sample runs. Furthermore, an association of z-score normalized ΔSF and ΔSpO_2_ was investigated using the Bland Altman and correlation analyses, which indicate a weak correlation (r = 0.15, *p* < 0.005) and poor agreement (95% limits of agreement LoA: [−2.6, 2.6]) for the derivation dataset. Their plots are illustrated in Fig 4.

**Fig 5:**
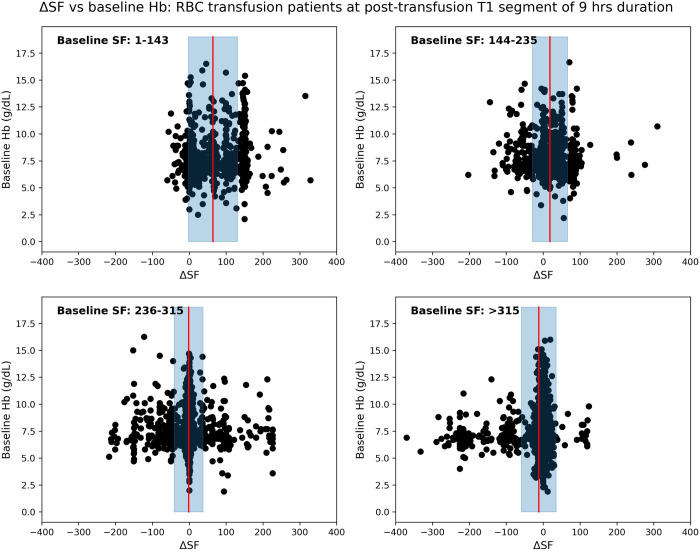
Localized study on Grady hospital patients having different ranges of baseline SF ratios. ΔSF vs baseline Hb for first post-transfusion region T1 of duration 9 hours with baseline SF range (a) 1-143, (b) 144-235, (c) 236-315, and (d) >315. Note that data-points denote the distribution of patients. Red vertical line with blueish filled region around it represents the mean ± standard deviation of the SF difference.

**Fig 6:**
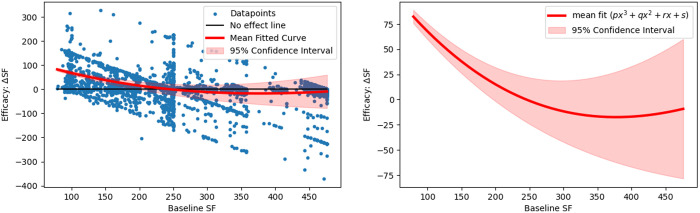
Validation data: Relationship between baseline SF (BSF) and transfusion efficacy (ΔSF). Resultant mean curve (solid red) and its 95% confidence interval (shaded red region) modelled with a cubic-polynomial curve fitting demonstrates an inverse relationship of BSF and ΔSF. Modeling parameters of mean fitted curve are as follows: −0.0000008, 0.0018, −1.007, 151.81.

**Fig 7:**
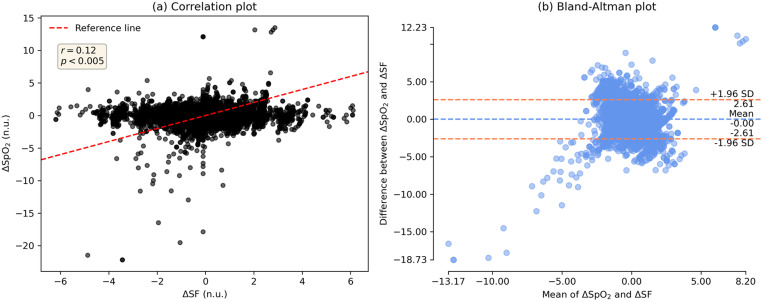
Bland Altman analysis of normalized ΔSF and ΔSpO_2_ on the validation data. (a) correlation plot, and (b) Bland-Altman plot.

To check the consistency and reproducibility of results, we validated our method with Grady Hospital data. Similar to the derivation data, Table 2 presents the distribution of transfused patients in four baseline SF groups for the validation data. For the first post-transfusion segment T1, the relation between ΔSF and baseline Hb is shown for all four baseline SF groups in Fig 5; whereas Supplemental eFig 2 in S1 File shows a detailed picture of this relationship for T1, T2 and T3. We observed that patients with the lowest baseline SF ratio (1–143) showed an improvement in SF ratio after RBC transfusion. While patients with a baseline SF > 315 demonstrated no significant improvement in post-transfusion SF ratios. These findings demonstrated a similar pattern that was obtained by the derivation data, ensuring the consistency. Additionally, the resulting modelled curve on ΔSF and baseline SF, depicted in Fig 6, showed an inverse relationship of them, which was also consistent. Like for the derivation data, we also attempted to show the association of ΔSF with ΔSpO_2_ using the Bland Altman analysis in Fig 7, which also yielded poor and similar correlation (r = 0.12, *p* < 0.005) and agreement (LoA: [−2.61, 2.61]).

The random forest regressor yielded R-squared values of 0.211 and 0.209, MSE of 2040.6 and 1868.84, and MAE of 23.89 and 23.1 for test and validation sets, respectively with significance (*p* < 0.001). Mean cross-validation MSE was obtained as 2015.81 in 10-fold model building process. The random forest regression was implemented on python with standard parameter settings of *sklearn* library.

## Discussion

In the present study, we demonstrated how strongly baseline SF, i.e., pre-transfusion SF ratio is associated with the RBC transfusion response, shown by ΔSF measurement, with substantial experiments on two different centers. Critically ill patients with physiologic derangements having lowest SF 1–143 benefitted more from the RBC transfusion. While respiratory physiology of population with the least respiratory compromise (baseline SF > 315) worsened subsequent to RBC transfusion. We observed that the results across both the centers were consistent. This initial study establishes SF as a potential marker to evaluate the RBC transfusion efficacy.

Alongside hemoglobin, post-transfusion SF difference (ΔSF) may be used effectively to measure the efficacy of RBC transfusion. Figs 2, 5 and eFigs 1, 2 in S1 File show a gradual decline of ΔSF for patient-groups as we move in their order from A to D. That means there existed almost linear and continuous decrement in post-transfusion SF change of patient-groups having incremental baseline SFs, and this effect is consistent throughout each of all three post-transfusion regions. Decrement in ΔSF signifies the degree of deterioration of transfusion efficacy. This effect was also shown via polynomial curve modeling approach. Also, the ΔSpO_2_ had poor agreement with ΔSF, highlighting that SpO_2_ alone may not reflect the changes in physiology informed by the more complete SF ratio.

The present study is first of its kind, specifically designed for critically ill patients in ICU. The conventional clinical practice has been over reliant on Hb [1,2,4], where an increase in post-transfusion Hb is expected in anemic patients. Additionally, from Tables 1 and 2, we observed a decreasing effect in baseline Hb and hematocrit ranges as we move from A to D population. Where patients in group D with highest baseline SF are least critical, characterized by smallest Sequential Organ Failure Assessment (SOFA) max total score, and patients in A with lowest baseline SF are most critical, reflecting large SOFA scores. Thus, an inability to uncover the impact on respiratory physiology subsequent to RBC transfusion makes Hb incapable to show the transfusion efficacy for ICU patients. To strengthen Hb-reliant conventional approach, our study introduced SF as a surrogate and showed that effectiveness of the RBC transfusion is strongly associated with the pre-transfusion SF ratio. However, future prospective studies and clinical trials may be required to validate the hypothesis for its more generalizability. The ΔSF measure can be used as a convenient assessment tool for transfusion efficacy, especially for anemic patients with chronic lung diseases and other respiratory physiological derangements. The best part of ΔSF is that it can be measured instantly within seconds unlike lab-values and non-invasively, providing convenience to the patients. Factors majorly affecting ΔSF include inaccuracies in SpO_2_ and FiO_2_ measurements. Our study has several limitations from the perspective of measuring SpO_2_ by the pulse oximetry method. First, accuracy of pulse oximetry decreases significantly when SpO_2_ falls below 80%, and also during poor peripheral perfusion in conditions like shock and vasoconstriction. Second, darker skin pigmentation and thicker skin can affect the accuracy of pulse oximetry readings. Third, movement of the patient or the sensor can cause inaccuracies in the SpO_2_ readings. Fourth, in occult hypoxemia cases, patients are hypoxemic despite having acceptable SpO_2_ readings. The SF ratios computed from erroneous SpO_2_ measurements can significantly affect the performance of this study. Additionally, number of transfusion units received by patients and their age-group information are potential confounding factors that were not considered in the current study when evaluating the effectiveness of RBC transfusion, thereby representing a potential limitation. We plan to examine the association and impact of these factors in the near future, as this will be a critical next step in enhancing the performance of the regression model.

Based on this initial study, we demonstrate that pre-transfusion SF ratio is inversely associated with RBC transfusion response, introducing SF ratio as a measure for RBC transfusion efficacy. Additionally, we show that SF ratio is not meaningfully correlated to SpO_2_ or Hb. Our results suggest that additional studies evaluating the SF ratio as a biomarker to determine the response to RBC transfusion (e.g., ΔSF) and the neediest RBC transfusion recipients (e.g., SF ≤ 143) are warranted. Our findings are consistent with studies showing the lack of improvement in oxygenation following RBC transfusion for many critically ill patients and emphasize the need for pragmatic, readily available measures to more precisely identify those critically ill patients who are most likely to benefit from RBC transfusion [12,13]. Further studies are needed to analyze the association of transfusion reactions (e.g., TACO, TRALI) in the SF groups to the physiologic deterioration.

## Supporting information

S1 File Supplemental digital content. The manuscript links a.docx file, supplemental_material.docx, that carries two supplemental figures (efigure 1 and efigure 2), referred in this manuscript.
(DOCX)
