# Supplementary material for: SpO2/FiO2 ratio as a better metric for assessment of RBC transfusion effectiveness in non-traumatic critically ill patients with physiologic derangements
Source: PLoS One. 2025 Jul 3;20(7):e0327537. doi: 10.1371/journal.pone.0327537 (PMC12225830; doi:10.1371/journal.pone.0327537)
Supplement: S1 File — The manuscript links a.docx file, supplemental_material.docx, that carries two supplemental figures (efigure 1 and efigure 2), referred in this manuscript. (DOCX) [file pone.0327537.s001.docx]

**Supplemental Digital Content**

**SpO_2_/FiO_2_ Ratio as a Better Metric for an Assessment of RBC Transfusion Effectiveness in Non-traumatic Critically Ill Patients with Physiologic Derangements**

**Authors:** Tilendra Choudhary *Ph.D.*^1,*^, Geoffrey Smith *M.D.*^2^, John D. Roback *M.D.*^2^, Ravi M. Patel *M.D.*^2^, Cassandra D. Josephson *M.D.*^2,3,4^, Rishikesan Kamaleswaran *Ph.D.*^1^

^1^Duke University School of Medicine, Durham NC.

^2^Emory University School of Medicine, Atlanta GA.

^3^Department of Oncology, The Johns Hopkins University School of Medicine, Baltimore, Maryland, USA.

^4^Cancer and Blood Disorders Institute, Johns Hopkins All Children’s Hospital, St. Petersburg, Florida, USA.

**Short title:** SF Ratio as a marker to evaluate RBC transfusion efficacy in critical illness

**E-mail addresses:** tilendra.choudhary@duke.edu (Tilendra Choudhary), geoffrey.smith@emory.edu (Geoffrey Smith), jroback@emory.edu (John D. Roback), rmpatel@emory.edu (Ravi M. Patel), cjoseph@emory.edu (Cassandra D. Josephson), r.kamaleswaran@duke.edu (Rishikesan Kamaleswaran)

***Corresponding author:** Tilendra Choudhary, e-mail: tilendra.choudhary@duke.edu

**Table of Contents**

1. eFigure 1 ……………………………………………………………………………….….3
2. eFigure 2. …………………………………………………………………….……………4

This supplementary document includes two figures with reference to the manuscript. eFigure 1 illustrates a localized study on Emory patients having different ranges of baseline SF ratios. After the transfusion event, a change in SF is measured and presented with baseline hemoglobin for three post-transfusion segments T1, T2 and T3. We observed that patients with the lowest baseline SF ratio (1-143), reflecting those most critically ill, consistently demonstrated an improvement in SF ratio subsequent to RBC transfusion. For patients with a baseline SF >315, reflecting the population with the least respiratory compromise, no significant improvement in SF ratio was observed following RBC transfusion, and in fact respiratory physiology worsened after transfusion. eFigure 2 illustrates the same study on Grady patients’ data for the validation purpose.

| 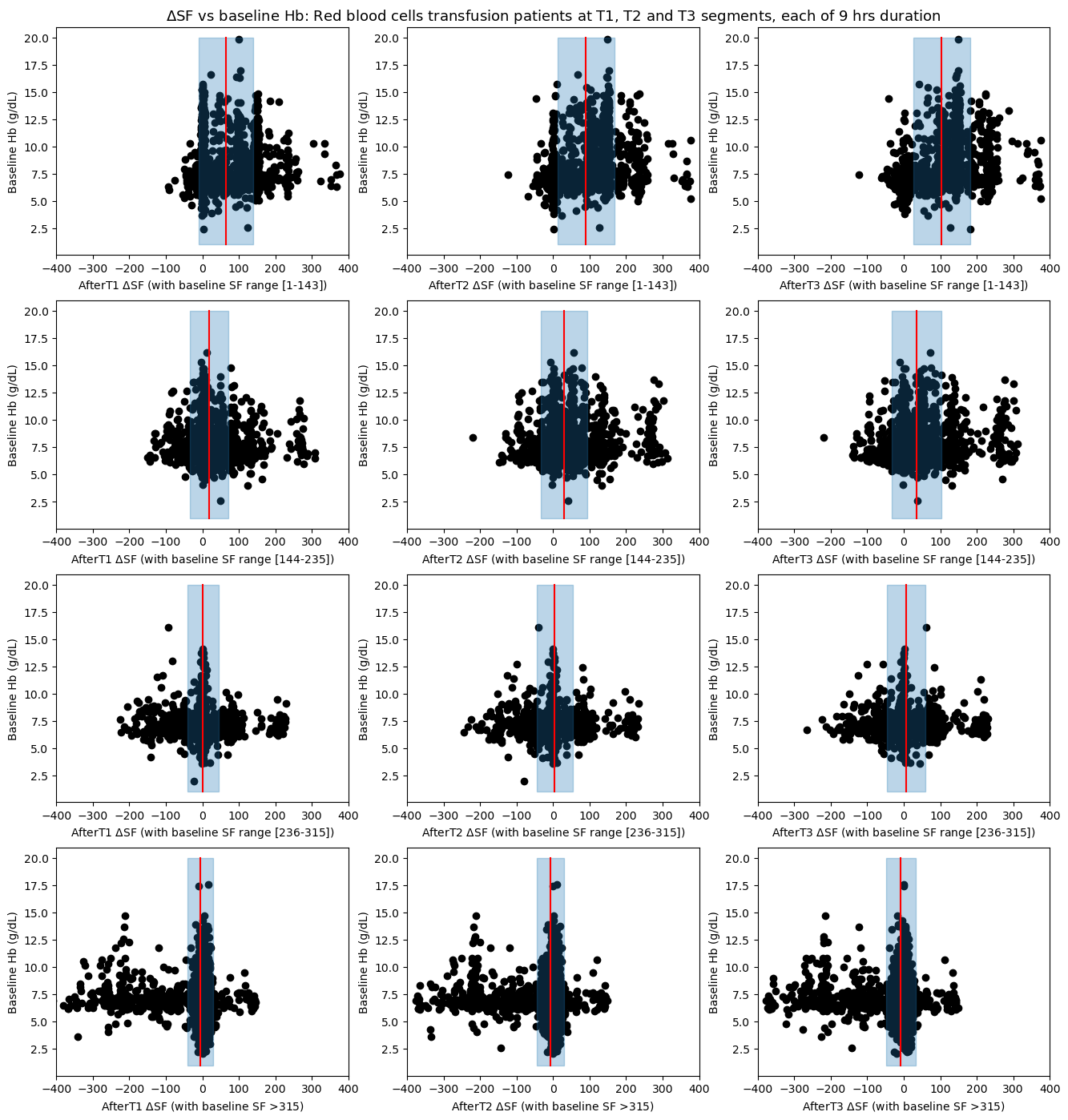 |
| --- |
| **eFigure 1:** **Emory data:** **Localized study on patients having different ranges of baseline SF ratios.** ΔSF vs baseline Hb for three consecutive post-transfusion regions (each of duration 9 hours) with baseline SF range [row-1] 1-143, [row-2] 144-235, [row-3] 236-315, and [row-4] >315. Note that data-points denote the distribution of patients. Red vertical line with blueish filled region around it represents the mean ± standard deviation of the SF difference. |

| 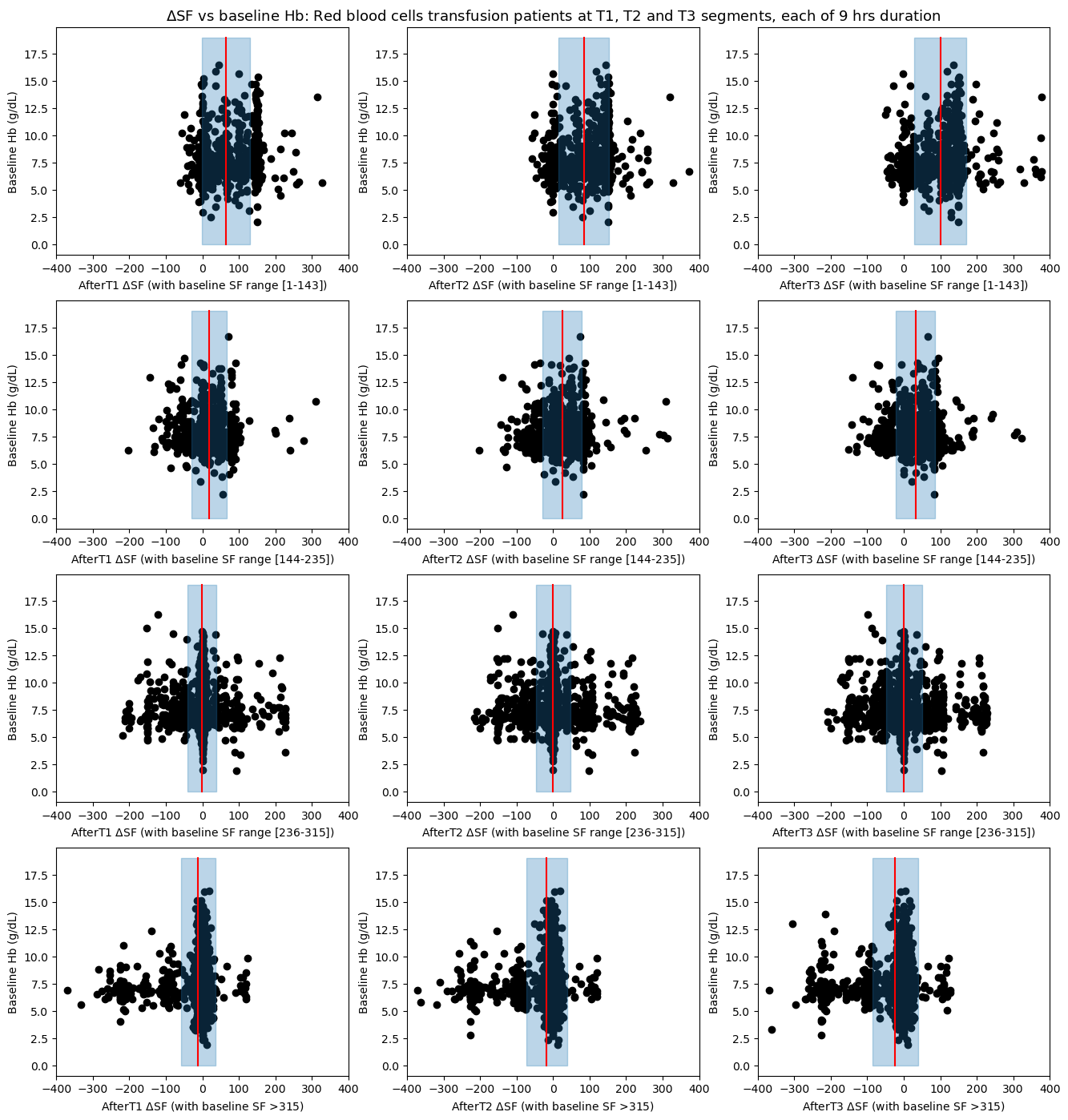 |
| --- |
| **eFigure 2:** **Grady data:** **Localized study on patients having different ranges of baseline SF ratios.** ΔSF vs baseline Hb for three consecutive post-transfusion regions (each of duration 9 hours) with baseline SF range [row-1] 1-143, [row-2] 144-235, [row-3] 236-315, and [row-4] >315. Note that data-points denote the distribution of patients. Red vertical line with blueish filled region around it represents the mean ± standard deviation of the SF difference. |
